# Functional Characterization of Glycated Peptide Aggregates in Whey Protein Hydrolysates

**DOI:** 10.1002/fsn3.4704

**Published:** 2025-02-19

**Authors:** C. J. Slingerland, M. Laats, H. F. J. Savelkoul, R. J. J. van Neerven, M. Teodorowicz

**Affiliations:** ^1^ Cell Biology and Immunology Group Wageningen University & Research Wageningen The Netherlands; ^2^ FrieslandCampina Amersfoort The Netherlands

**Keywords:** basophil degranulation, glycated aggregates, Maillard reaction, RAGE, whey protein hydrolysates

## Abstract

Heating food proteins promotes a reaction between proteins and sugars called the Maillard reaction (MR). Maillard reaction products (MRPs) have been linked to increased immunogenicity of proteins through interaction with receptors for advanced glycation end products (RAGE). Here, we aimed to characterize the functional properties of whey protein hydrolysates (WPHs) and its' fractions. A partial WPH1 and an extensive WPH2 were size fractionated. The MRPs were detected with anti‐Nε‐carboxymethyllysine (CML) antibody and binding to RAGE was measured using inhibition ELISA. Induction of pro‐inflammatory cytokines was determined in THP‐1‐derived macrophages, and the capacity to induce degranulation of basophils was assessed using FcεRI+ RBL cells. The partial WPH1, but not WPH2, contained high MW fractions (aggregates > 100 kDa) which bound to RAGE and induced the production of IL‐6, IL1‐β, and IL‐8 in THP‐1 macrophages. The aggregates of WPH1, but not the smaller fractions, induced the degranulation of FcεRI+ RBL cells. The presence of high MW glycated aggregates in partial WPHs leads to increased binding to RAGE, production of pro‐inflammatory cytokines, and basophil degranulation in the presence of whey‐specific IgE. This implies that the safety and functionality of partially hydrolyzed formulas should not be generalized due to their composition and potential immunogenicity of glycated aggregates.

## Graphical abstract

Cow's milk‐derived whey proteins are used extensively for infant formulas. During processing steps (at elevated temperature whilst lactose is present), Maillard reactions and peptide aggregation take place, affecting the immunogenic potential of the resulting whey peptides. The glycated aggregates but not the low molecular weight fractions bind to receptor for advanced glycated end products (RAGE) and induce degranulation of FcεRI+ RBL cells loaded with serum IgE from cow's milk allergic individuals.

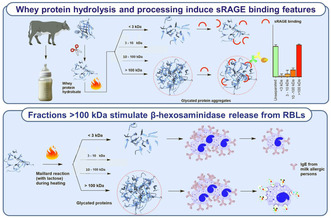

**Abbreviations**
AGEs: advanced glycation end products
BLG: β‐lactoglobulin
CMA: cow's milk allergy
CML: N^ε^‐carboxymethyllysine
HFs: hydrolyzed cow's milk infant formulas
MR: Maillard reaction
MRPs: Maillard reaction products
RAGE: receptor for advanced glycation end products
WPHs: whey protein hydrolysates

## 1 Introduction

Immunoglobulin E (IgE)‐mediated cow's milk allergy (CMA) is one of the most common food allergies in early life (Flom and Sicherer 2019). A first choice for the treatment of CMA in infants according to the guidelines of European Academy for Allergy and Clinical Immunology (EAACI) should be hydrolyzed cow's milk infant formulas (HFs) with documented hypoallergenicity (Muraro et al. 2014). Bovine whey proteins are the most common source of proteins in the production process of HFs. The production process itself is complex and involves several heating steps such as sterilization, spray‐drying, or/and ultrahigh temperature treatments. A heat treatment in the presence of carbohydrates promotes a number of protein modifications including denaturation, aggregation, and modification by Maillard reaction (MR). The MR occurs between a free amino group of protein, mostly lysine, and a carbonyl group of reducing sugar, in this case lactose that is naturally present in milk (Pischetsrieder and Henle 2012; Teodorowicz, Van Neerven, and Savelkoul 2017). A consequence of the MR is a series of chemical rearrangements leading to the formation of structurally diverse classes of molecules called advanced glycation end products (AGEs). Formation of AGEs in infant formulas has been well documented as well as its effect on lysine blockage and impaired protein digestibility (Pischetsrieder and Henle 2012; Zenker, van Lieshout, et al. 2020). The level of MR in food and infant formulas is determined based on the analysis of well‐characterized AGEs such as N^ε^‐carboxymethyllysine (CML) (Prosser, Carpenter, and Hodgkinson 2019). Importantly, the formation of AGEs enhances the immunogenicity of proteins by specifically binding to several receptors present in innate immune cells. The best‐studied interaction of AGEs with the immune system is via the receptor for advanced glycation end products (RAGE). The interaction with RAGE has also been shown for glycated whey protein aggregates (Liu et al. 2016). Activation of RAGE leads to an intracellular signaling cascade, resulting in the induction of pro‐inflammatory cytokines, inflammation, and oxidative stress. Recently, it has been shown that the larger fractions (> 100 kDa) of heated β‐lactoglobulin (BLG) aggregates are predominantly responsible for the binding to the RAGE, as well as to CD36, SR‐AI, and Galectin‐3 (Teodorowicz et al. 2021). Similarly, cross‐linked BLG was increasingly endocytosed by dendritic cells (DCs), in a murine food allergy model. Apart from the immunogenic potential of BLG aggregates, several studies indicate that they may also have increased allergenicity. Endocytosis of cross‐linked BLG by DCs led to Th2‐stimulating DCs and specific IgG1 and IgE production (Stojadinovic et al. 2014). The Th2‐associated environment was also seen as a result of pasteurization‐induced aggregates of BLG and alanine (ALA) in sensitized mice (Roth‐Walter et al. 2008).

Still little is known about the possible effect of glycation and aggregation on the functional characteristics of hydrolyzed milk proteins, more specifically milk hydrolysates generated in the presence of lactose. To this aim, we investigated whether whey protein hydrolysates (WPHs) fractions and aggregates can bind to RAGE, stimulate cytokine production, and whether they are able to induce the degranulation of basophils.

## 2 Materials and Methods

### 2.1 Characterization of WPHs

WPHs were obtained from spray‐dried whey protein concentrate (WPC) and provided by Friesland Campina (Wageningen, The Netherlands). The partial hydrolysate WPH 1 contains: 73.3% protein, 5.7% fat, 1.9% lactose, and < 5% water, while the filtered extensive hydrolysate WPH 2 contains 78.2% protein, 4.1% lactose, 0.1% fat, and 4.0% water. The degree of hydrolysis of WPH 1 was estimated to be around 18% while for WPH 2 it was 27.6%. The LPS concentration in WPHs was measured using the commercially available Endozyme Recombinant Factor C assay (Hyglos, cat. # 609050) according to the protocol of the manufacturer.

### 2.2 Preparation of Samples

Hydrolyzed, spray‐dried WPH 1 and WPH 2 were dissolved in MilliQ at a protein concentration of 50 mg/mL and centrifuged (2000 g, 10 min, RT), followed by incubation at 4°C for 15 min to allow separation of the fat from the aqueous layer. The soluble fraction was collected and the protein concentration was determined with UV absorbance (A280) measured by a Nanodrop system (ThermoFisher Scientific, Waltham, USA).

### 2.3 Fractionation of Samples

To study protein size‐dependent effects, WPH 1 and WPH 2 dissolved in the aqueous layer were fractionated in size‐based fractions using Amicon Centrifugal Filter Units (Merck Millipore, Billerica, MA, USA). The protein solution was first loaded into an Amicon 100 kDa Filter Unit (#UFC910008; Merck Millipore, Billerica, MA, USA) followed by centrifugation (3300 g, RT, 10 min). The fraction remaining in the filter was re‐suspended in MilliQ and the centrifugation was repeated five to seven times. Subsequently, the combined flow‐through fractions were fractionated in the same way using 10 and 3 kDa Filter Units (#UFC901008 and #UFC900308; Merck Millipore, Billerica, MA, USA). Finally, the following fractions were obtained: molecular weight (MW) < 3 kDa, MW 3–10 kDa, MW 10–100 kDa, and MW > 100 kDa. The accuracy of the fractionation was assessed by size exclusion chromatography (SEC) with an ACQUITY UPLC Protein BEH SEC Column 125 Å, 4.6 × 300mm prior by a Guard column, and 4.6 × 30mm (Waters Corp., Milford, MA, USA) using UPLC system (Waters Corp., Milford, MA, USA). Phosphate buffer (PBS, 100 mM; 150 mM NaCl, pH 6.8) was used for elution with the isocratic flow rate of 0.3 mL/min. The elution of the proteins was monitored at 214 nm.

### 2.4 Detection of CML Using a Chemiluminescent Dot‐Blot Immunoassay

The proteins (WPH 1 and WPH 2 or their fractions) were spotted in 0.45 μm nitrocellulose membrane (GE Healthcare Life science, Marlborough, Massachusetts, USA). After drying, the membrane was blocked for 1 h at room temperature with 3% BSA in TBST (50 mM Tris, 150 mM NaCl, pH 7.5). Subsequently, membranes were washed 2 × 10 min with TBST with 0.05% Tween‐20 and incubated at 4°C overnight with mouse anti‐CML antibodies (IgG1; Biologo #CML011) diluted in TBS with 1.5% BSA. The next day, the membrane was washed extensively with TBST/Triton and subsequently incubated with anti‐mouse polyclonal goat HRP conjugated antibody (Dako, # PO447) diluted 1:1000 in TBST with 1.5% BSA. After incubation, membranes were washed with TBST/Triton and lastly with TBS and developed with ECL western blot detection reagent (Advansta; K‐12031‐001; San Jose, USA) for 30 s. Chemiluminescence was visualized in ChemHighsensitivity mode using a Universal Hood III (Bio‐Rad, Hercules, California, USA) and Image Lab 4.1 software (Bio‐Rad, Hercules, California, USA).

### 2.5 sRAGE Binding Assay

To determine the binding affinity of WPHs to recombinant soluble RAGE (sRAGE), the competition ELISA assay was performed, as described before (Teodorowicz et al. 2021). sRAGE (Soluble Advanced Glycation End Product‐Specific Receptor, Human *E. coli*; cat no RD172116100, BioVendor, Brno, Czech Republic) at the concentration of 1.25 μg/mL was used and WPHs at the following dilutions: 2.5; 25; 250; and 2500 μg/mL. Results were expressed as a percentage of inhibition with reference to the maximum signal (sRAGE at 1.25 μg/mL). Ovalbumin (# vac‐stova, Invivogen, Toulouse, France) was used as a negative control.

### 2.6 THP‐1 Assay

The human monocytic leukemia cell line THP‐1 (American Type Culture Collection, Rockville, Md.) was cultured in RPMI 1640 with l‐glutamine and 25 mM HEPES, supplemented with 10% FBS and 1% penicillin/streptomycin at 37°C and 5% CO_2_ in a humidified incubator. Macrophage differentiation was induced by treatment of THP‐1 monocytes (10^6^ cells/mL) for 48 h with 10 ng/mL phorbol 12‐myristate 13‐acetate (PMA, cat. # P1585; Sigma) in 96‐well cell culture plates containing 100 μL of cell suspension. After 48 h, the cells were washed twice with medium and incubated for another 48 h. Next, the THP‐1 macrophages were stimulated for 24 h with various dilutions of WPHs diluted in RPMI 1640.

FPS‐ZM1 (Merck Millipore, # 553030) was used as a RAGE inhibitor at a final concentration of 3.0 μM; After 24 h of incubation, the supernatant was collected and human cytokine concentrations (IL‐6, IL‐8, IL‐1β, IL‐10, and TNF‐α) were determined using the cytometric bead array (CBA) kit (Human Inflammatory Cytokine Kit, BD Bioscience, cat. # 551811) according to the manufacturer's instructions. The samples were analyzed by flow cytometry (BD FACS Canto II, BD Bioscience).

### 2.7 Mediator Release Assay (MRA) Using Rat Basophilic Leukemia Cells

The IgE‐dependent capacity of milk hydrolysates to degranulate basophils was tested on rat basophil leukemia cells (RBLs), as described before (Vissers et al. 2011). RBLs were grown in MEM with 5% FBS and 1% glutamine at 37°C, 5% CO_2_. Cells were grown to around 100% confluency and seeded in 96 well plates at a density of 7.5 × 10^4^/well. Human serum from cow's milk allergic patients was received from Rijnstate Hospital (Department of Clinical Chemistry and Hematology, Rijnstate Hospital, Arnhem, The Netherlands). Equal volumes of the serum obtained from three clinically documented milk‐allergic patients (Table S1) were pooled and the level of IgE specific for cows' milk was estimated to be 132 kU IgE/L. To prevent potential interference with cellular assays, IgG was removed from the serum with a commercially available IgG removal kit (#89979; ThermoFisher Scientific, Waltham, USA) in accordance with the manufacturer's protocol. The IgG‐depleted human serum from cow milk allergic patients (60× diluted) was added to the RBL‐2H3 cells. For positive controls, the cells were incubated with IgE (100 ng/mL, # 7050; Diatec AS, Norway) in MEM. After 24 h of incubation at 37°C, the cells were washed gently three times with 75 μL Tyrode's washing buffer. After washing, whey hydrolysate samples were added to the cells (100 μL/well). Positive controls were incubated with anti‐IgE (1.0–32 μg/mL, # GAHu/IgE(Fc)/7S, Nordic‐MUbio, The Netherlands). After 1 h of incubation (37°C, 5% CO_2_), 60 μL of the supernatant of the cells was incubated with 50 μL substrate solution (3.80 mM p‐nitro‐N‐acetyl‐β‐d‐glucosaminide, Sigma–Aldrich, # N9376) for 1 h at 37°C. Cells, treated with Triton‐X100 (1% in PBS, 100 μL) served as a positive control for the total β‐hexosaminidase release capacity of the RBLs. The reaction was stopped by the addition of 100 μL glycine (0.2 M, pH 10.7). The absorbance was measured at 405 nm with 620 nm as reference using FilterMax F5 Plate Reader (Molecular Devices, California, USA). Release data are corrected for the release in the absence of a hydrolysate sample and expressed as relative release.

### 2.8 Statistical Analysis

Results were analyzed by ANOVA (one‐way analysis of variance), followed by a Tukey post‐test. All analyses were carried out in Graphpad Prism software (version 5.03). Data are shown as average + standard deviation. In all cases, the level of significance is indicated by stars, *p ≤ 0.05; **p ≤ 0.01; ***p ≤ 0.001.

## 3 Results

### 3.1 Characterization of WPHs

**FIGURE 1:**
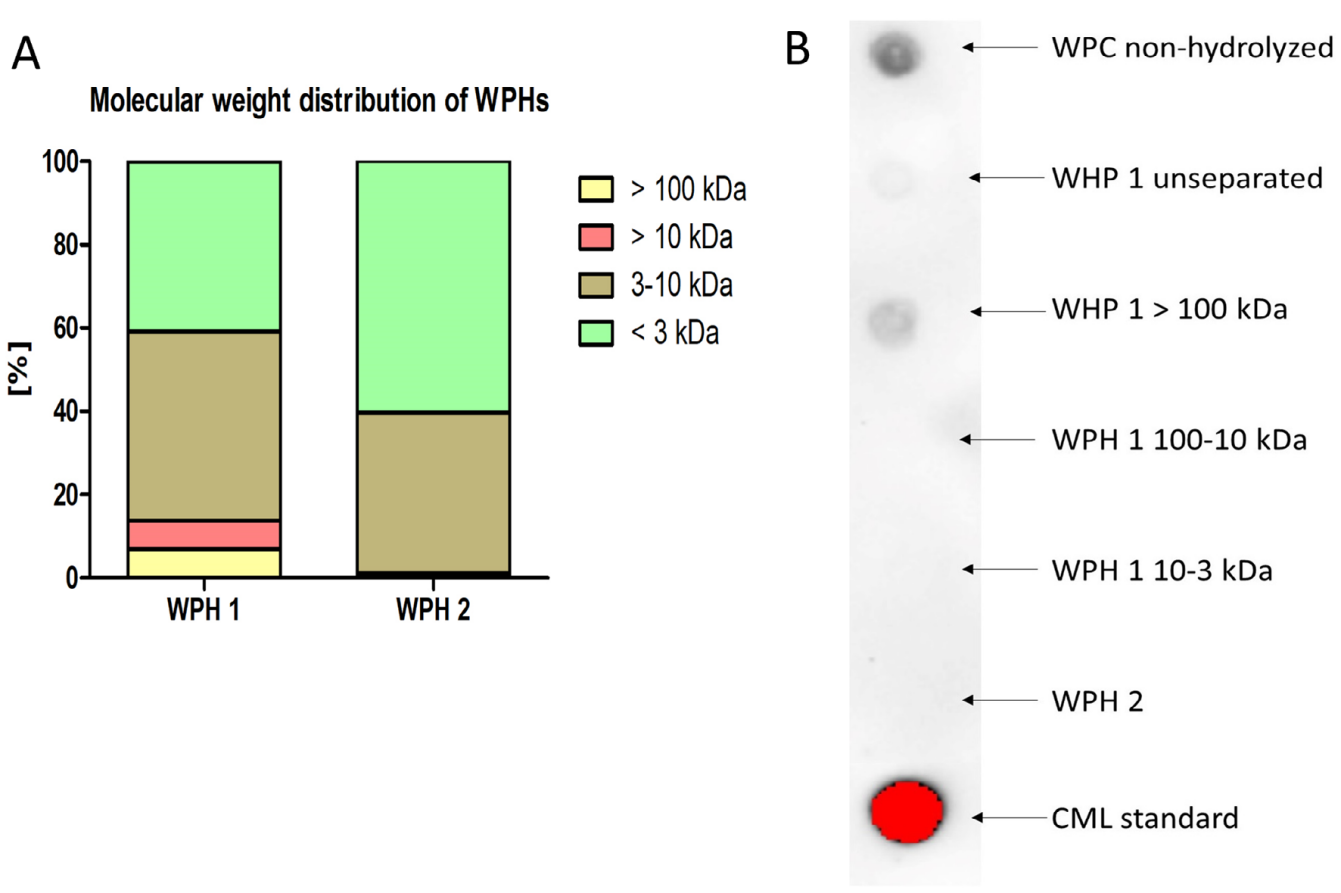
Characterization of WPHs. (A) Molecular weight distribution in each of WPH measured by UPLC. (B) CML content in the fractions obtained from WPH 1 and in unseparated WPH 2 detected with dot‐blot; Positive control—CML standard; WPC non‐hydrolyzed whey protein extract.

In this study, the partial WPH1 obtained from spray‐dried WPC with a degree of hydrolysis of 18% was compared to extensively hydrolyzed WPH2 with a degree of hydrolysis of 28%. WPH2 was additionally filtered during the production process to remove high MW fractions. The LPS contamination in each WPH was determined to be below 5 pg/mL. The MW distribution of each WPH was analyzed using UPLC (Figure 1A). WPH 1 was composed mostly of small peptides of 3–10 kDa (45%) and < 3 kDa (41%). However, also larger peptides were detected with MW higher than 10 kDa (7%) as well as components of > 100 kDa, possibly aggregates (7%). The distribution of MWs of WPH 2 differed from WPH 1 and was dominated by low MW peptides (below 3 kDa, 60%) and peptides with a MW of between 3 and 10 kDa (39%). The content of the large peptides of 10–100 kDa and > 100 kDa was estimated to be below 1%.

To study the correlation between the peptide/protein size and the functionality/immunogenicity of these WPHs, the samples were fractionated using Amicon Centrifugal Filter Units to obtain four different fractions: MW < 3 kDa, MW 3–10 kDa, MW 10–100 kDa, and MW > 100 kDa. The accuracy of the sample separation in MW‐based fractions was controlled by size exclusion chromatography (Figure S1). The purity of the fraction of < 3 kDa was estimated to be 67% ± 1.1 whereas for the fraction of MW 3–10 kDa and > 10 kDa, the accuracy was 82 ± 0.7 and 70 ± 7.7, respectively. Taken together, these data show that the method of MW‐based protein separation of complex whey protein mixtures by Filter Unit centrifugation gives an accuracy of 73% ± 6.5 and that the fractions of interest are the main components in these fractions (Figure S1).

Importantly, our current method of fractionation does not induce additional aggregation. As the unseparated WPH 2 contains hardly proteins > 10 kDa, the separated fractions contain hardly proteins > 10 kDa (Figure S1).

To assess the glycation level in the fractionated WPH 1 as well as unseparated WPH 2 CML was detected with chemiluminescence dot‐blot immunoassay by the use of anti‐CML antibodies. As presented in Figure 1B the highest content of CML was detected in the fraction with the MW higher than 100 kDa isolated from WPH 1, while a weak signal was detected in the unseparated WPH. The content of CML in the fractions with MW below 100 kDa and WPH 2 was below the detection limit of the method. These data indicate a higher level of MR (glycation) of the aggregated fractions with MW higher than 100 kDa.

### 3.2 Enhanced Binding of Large WPH Fractions to Soluble Receptor for Advanced Glycation End Products (sRAGE)

**FIGURE 2:**
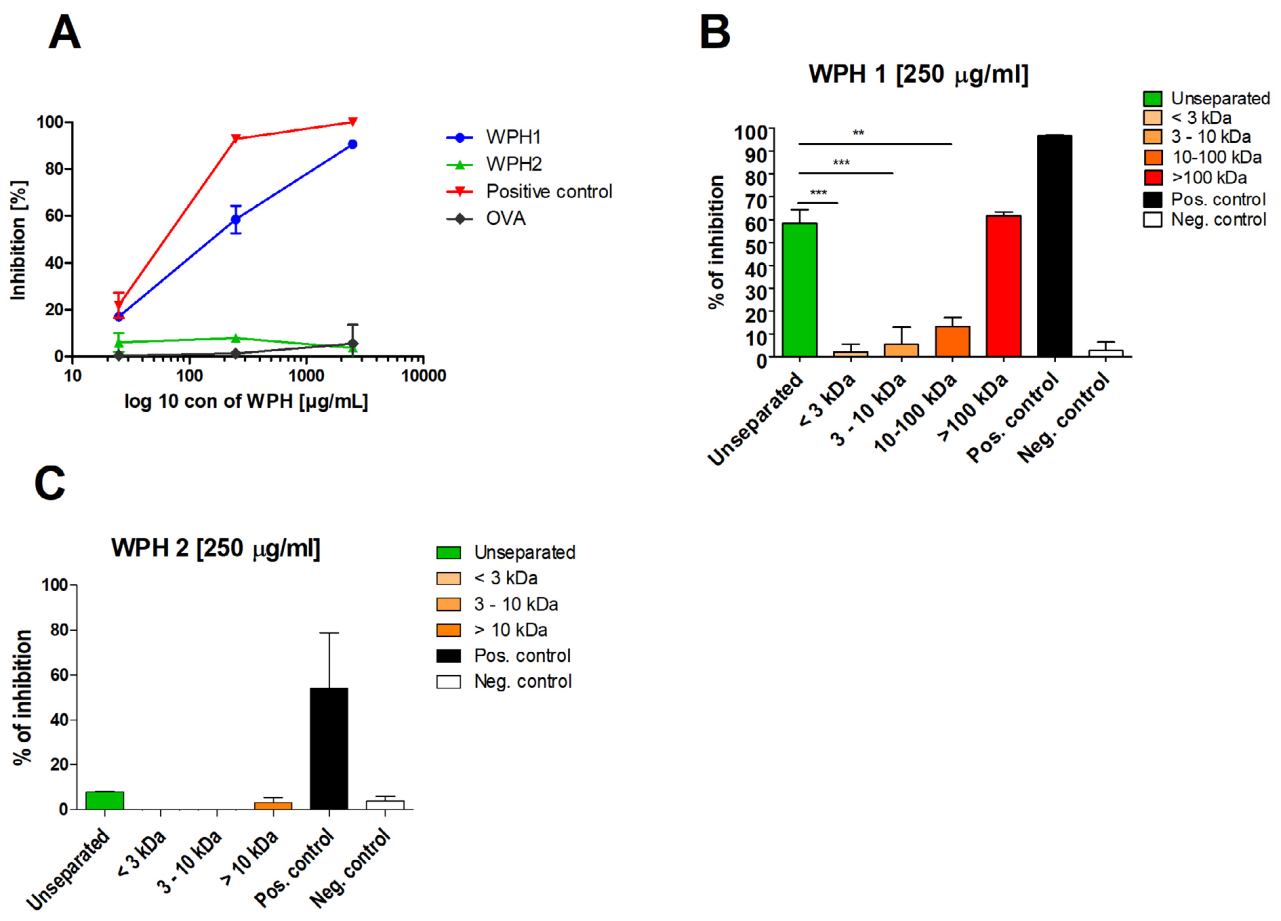
Inhibition of sRAGE by WPHs before fractionation (A) and after fractionation (B, C) measured in competition ELISA. WPHs were pre‐incubated with the recombinant form of sRAGE prior to adding to the ELISA plate coated with glycated soy protein extract. The data are expressed as a percentage of inhibition by the use of a non‐inhibited signal as the maximum value. Positive control: glycated soy proteins Negative control: ovalbumin. The graph shows an average from three independent experiments. Significant differences analyzed with one‐way ANOVA with Tukey post hoc comparison test (GraphPad Prism); **p < 0.01, ***p < 0.001.

Unseparated as well as fractionated WPHs were tested for their ability to bind to sRAGE in an inhibition ELISA. In this assay, sRAGE is allowed to bind to highly glycated soy proteins coated onto the bottom of the well, and the inhibition of this binding by the WPHs is measured. WPH 1, but not WPH 2, showed a concentration‐dependent inhibition of sRAGE binding (Figure 2A). Comparing the dose‐dependent inhibition curves of both WPHs to negative control (ovalbumin) WPH 1 showed significantly higher inhibition for all tested concentrations, whereas WPH 2 showed comparable inhibition to the negative control (Figure 2A). Most remarkably, very low inhibition was observed for WPH 2 which does not contain high MW fractions. To further investigate the sRAGE binding capacity of the high MW fractions versus the low MW fractions, both WPHs were fractionated and the fractions were analyzeds by sRAGE ELISA. Comparing the inhibitory capacity of the different MW fractions tested at 250 μg/mL clearly showed that the fractions with a lower MW than 100 kDa hardly contribute to the inhibition. In contrast, the fractions containing large components with a MW above 100 kDa showed an inhibition that is similar to the inhibition observed for the “total soluble proteins” of the unseparated WPH. Fractions obtained from WPH 2 were not recognized by sRAGE (Figure 2C). Summarizing, the binding of WPHs to sRAGE is explained by the presence of high MW protein aggregates.

### 3.3 Cytokine Release by THP‐1 Macrophages Stimulated With WPH 1 and WPH 2

**FIGURE 3:**
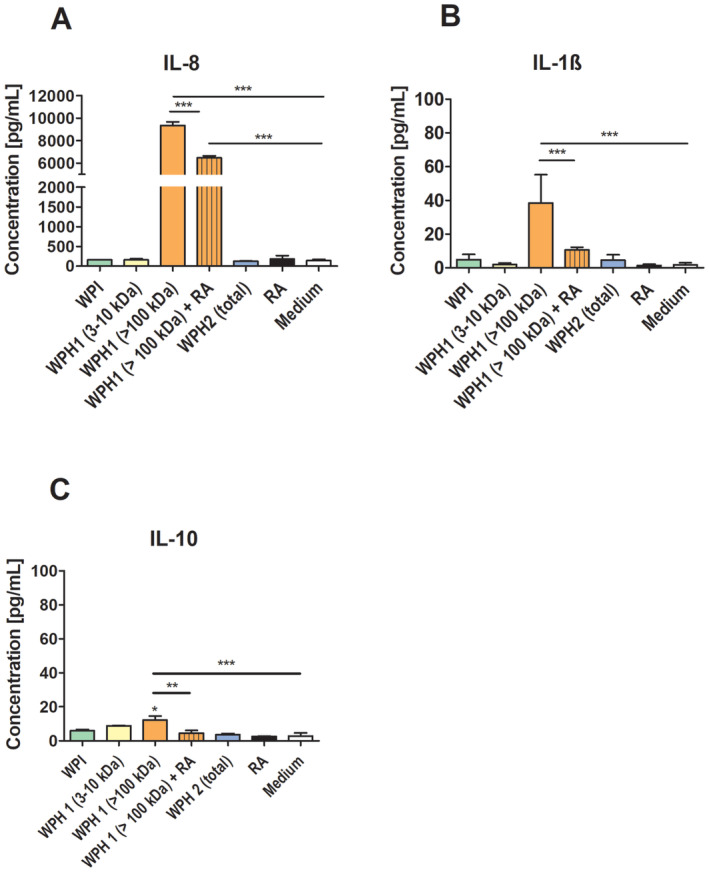
THP‐1 cytokine release upon stimulation with low and high molecular weight fractions obtained from WPH 1 and unseparated WPH 2 (total). Cells were incubated for 24 h with fractions obtained from WPH 1 and unseparated WPH 2 (total) without the presence of the RAGE antagonist FPS‐ZM1 (RA) and in the presence of RAGE antagonist FPS‐ZM1 (+RA). The cytokines levels in the supernatants were determined using the cytometric bead array (CBA) kit. Significant differences analyzed with one‐way ANOVA with Tukey post hoc comparison test (GraphPad Prism); *p < 0.05; **p < 0.01, ***p < 0.001. The levels of TNF‐α and IL‐6 were not significantly affected (data not shown). The graph shows an average from two independent experiments each performed in triplicates.

The monocyte‐derived cell line THP‐1 was used to assess activation of innate immune cells by WPH 1 and its' fractions and to compare it to compare to an activation caused by WPH 2 (unseparated). Figure 3 shows the levels of pro‐inflammatory cytokines IL‐1ß and IL‐8 as well as anti‐inflammatory IL‐10 in the medium after 24 h incubation of the fractions obtained from WPH 1 and WPH 2 with the THP‐1 macrophages. The levels of TNF‐α and IL‐6 were not significantly affected by the incubation of THP‐1 cells with WPHs (data not shown). The 3–10 kDa fraction of WPH 1 did not enhance the excretion of both IL‐8 and IL‐1ß compared to medium control. The same holds for unseparated sample WPH 2, which consists mostly of components smaller than 10 kDa (Figure 1). In contrast, fractions with MW > 100 kDa of WPH 1 enhanced secretion of IL‐1ß and IL‐8 to levels that were significantly higher than in the cells incubated with medium only. For IL‐10, only a significant increase was observed for WPH 1 (> 100 kDa). Yet, the relative increase is lower than observed for IL‐8 and IL‐1β.

The higher cytokine levels in the medium of the THP‐1 macrophages incubated in the presence of the fractions with MW > 100 kDa correlate well with both CML content (Figure 1B) and sRAGE binding (Figure 2B). Upon co‐incubation with RAGE antagonist FPS‐ZM1, both the IL‐8, IL‐1β, and IL‐10 excretion are significantly lowered (Figure 3). Whereas the reduction in IL‐10 brings it down to control levels, this is not the case for IL‐8 and IL‐1β. It suggests that RAGE‐mediated cytokine production plays a role among other non‐RAGE‐mediated mechanisms.

### 3.4 High MW WPH Fractions of > 100 kDa Stimulate β‐Hexosaminidase Release From RBLs

**FIGURE 4:**
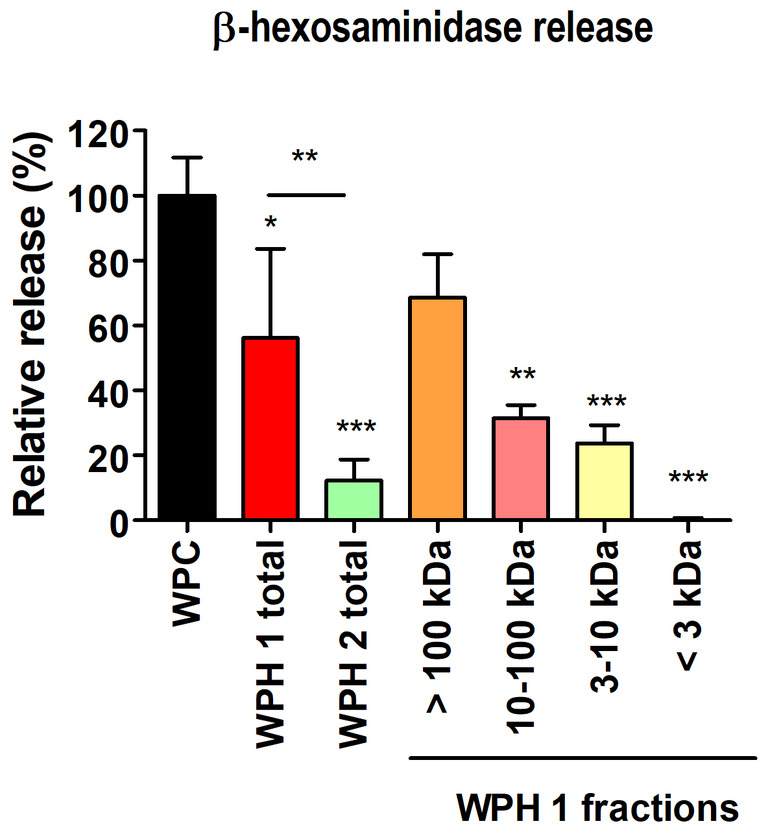
β‐hexosaminidase release from Rat Basophilic Leukemia Cells (RBLs) induced by WPH 1 and WPH 2. RBLs were incubated with 60× diluted human serum depleted of IgG, for 24 h, followed by washing and incubation with unseparated WPH 1 or WPH 2 (total) or different fractions of WPH 1 for 1 h. The spontaneous release has been subtracted from all values. The absolute release values have been standardized with reference to the release of WPC (non‐hydrolyzed whey protein concentrate). Significant differences analyzed with one‐way ANOVA with Tukey post hoc comparison test (GraphPad Prism); *p < 0.05; **p < 0.01, ***p < 0.001. If not indicated, the significant differences between WPC and each sample are depicted. The graph shows an average from three independent experiments each performed in triplicates.

Rat basophilic leukemia cells (RBLs) have been employed to assess the capacity of fractionated WPH1 as well as WPH2 (unseparated) to induce degranulation measured as β‐hexosaminidase release, as a quantifiable endpoint read‐out for degranulation. Stable expression of the human FcεRI receptor on those RBLs allows for interaction with human IgE, as isolated from cows' milk‐allergic individuals. Pooled IgG‐depleted serum of three cow‐milk allergic patients was used of which the sIgE levels are shown in Table S1. The data referred to the β‐hexosaminidase release caused by non‐hydrolyzed WPC as a positive control (Figure 4). The test was validated using the pooled sera and anti‐IgE in a series of dilutions prior to using WPC and the WPHs as degranulation agents.

WPH 1 induced a β‐hexosaminidase release which was significantly lower than the release induced by WPC and significantly higher compared to WPH2. WPH 2, induced significantly lower β‐hexosaminidase release than both WPC and WPH 1. To assess the difference in mediator release, induced by WPH 1 and WPH 2 in more detail, several fractions of WPH 1 were assessed individually. The high MW fraction of WPH 1 (> 100 kDa) induced comparable mediator release as unseparated WPH 1—being not significantly different from the release induced by WPC. Fractions with lower MW (all fractions below 100 kDa) as well as unseparated WPH 2 showed significantly lower mediator release compared to WPC which was positively correlated with the MW of the fraction. No release was induced by the fraction with MW < 3 kDa.

## 4 Discussion

In this study, we characterized differently processed WPHs and their size‐separated fractions obtained and evaluated their effect on innate immune cells and basophils. Partially, but not extensively, WPHs were shown to contain glycated aggregates with increased binding to the sRAGE compared to low MW protein fractions, which led to RAGE‐induced cytokine production. Strikingly, the high MW fraction, but not the low MW fractions, also showed the potential to lead to degranulation of FcεRI+ RBL cells loaded with sera of cow's milk allergic patients containing high levels of cow's milk allergen‐specific IgE.

Extensively hydrolyzed milk protein formulas (EHFs) are used to manage cow's milk allergy in very young non‐breastfed children. These hydrolysates mainly consist of very small peptides, that cannot induce mast cell release and thus prevent the occurrence of allergic reactions when they are ingested (Zepeda‐Ortega et al. 2021). Partially hydrolyzed milk protein formulas (PHFs), and sometimes EHFs, are used in children that are at risk of developing cow's milk allergy. However, there are significant differences in the peptide profiles and size distribution between commercially available EHFs and PHFs (Lambers et al. 2015) and even EHF can contain peptides or protein residues that are large enough to induce IgE binding and clinical reactions to ingestion of EHF (Chauveau et al. 2016; Nutten et al. 2020). In addition, casein hydrolysates can contain whey protein‐derived peptides and vice versa (Lambers et al. 2015). This indicates that various hydrolysates of the same type (e.g., EHF or PHF) may differ considerably in peptide composition and size, possibly helping to explain the differences in clinical outcomes seen in allergy prevention studies (Szajewska and Horvath 2010; Boyle et al. 2016). Here, we provide information that the presence of glycated aggregates in PHFs may be an additional factor that should be monitored.

The aggregation behavior of whey proteins has been studied extensively, both in mixtures of proteins (Nicolai, Britten, and Schmitt 2011) and in single proteins (Gulzar et al. 2013; Yan et al. 2013). Aggregation is a consequence of the heat‐induced denaturation of proteins which exposes reactive sites of amino acids for intra‐ and/or intermolecular interactions. A physicochemical characteristic of aggregates depends on a number of processing conditions including temperature and duration of treatment, pH, the presence of sugar, and other matrix compounds, as well as the water activity in the system. A high water activity seems to play an important role by facilitating the formation of aggregates over the formation of AGEs as it has been shown recently by Zenker and colleagues in a β‐lactoglobulin model (Zenker et al. 2019). On the other hand, low water activity (dry conditions that occur during spray drying) promotes the MR and the formation of AGEs (Liu et al. 2016) (Zenker et al. 2019). Therefore, water activity together with other processing conditions affect the physicochemical nature of the aggregates as is reflected in their size, hydrophobicity, and charge but also their susceptibility to enzymatic digestion, as well as their immunogenicity and allergenicity (Zenker, van Lieshout, et al. 2020; Teodorowicz et al. 2021; Bogahawaththa, Chandrapala, and Vasiljevic 2017; Bu et al. 2009). For instance, Leeb and colleagues showed that aggregation of β‐lactoglobulin, induced by thermal treatment, influences the release of functional peptides during enzymatic digestion (Leeb et al. 2015). This has been confirmed by Zenker et al. who analyzed peptide release from infant formulas with different levels of blocked lysine (from 6.5% up to 44.5%) during digestion in an in vitro infant digestion model. This study showed a higher number of longer peptides released from the infant formulas with the highest levels of blocked lysine, indicating that MR of milk proteins in an infant formula impairs overall protein digestibility (Zenker, van Lieshout, et al. 2020).

In the present study, we show that partial WPH obtained from spray‐dried WPC contains fractions with a MW higher than 100 kDa. Spray drying is known to induce aggregation and decrease solubility (Anandharamakrishnan, Rielly, and Stapley 2008). As WPHs also contain some lactose, spray drying may also affect the formation of MR products. The fraction with MW higher than 100 kDa was characterized by a higher content of CML compared to the fractions with lower MW indicating the involvement of MR in the formation of high MW structures/aggregates. CML is considered a good marker of the advanced stage of MR (Martins, Jongen, and Van Boekel 2000) and recently it has been shown to bind to the AGEs recognizing receptors, RAGE, Galectin‐3, and scavenger receptor class A, indicating the immunogenic character of this compound (Zenker, Teodorowicz, et al. 2020). In addition, the MR was previously shown to promote cross‐linking and aggregation of milk proteins (Corzo‐Martínez et al. 2010). Aggregates of BLG formed in the presence of glucose were shown to degrade much slower during gastrointestinal digestion compared to aggregates of control BLG heated without sugar, which were degraded much more rapidly (Zhao et al. 2019; Corzo‐Martínez et al. 2010). In the current study, the fraction with high MW > 100 kDa obtained from WPH 1 was shown to have the highest CML content as well as the highest sRAGE binding capacity. This finding is in line with the outcomes of previous research showing that aggregates formed during the heating of milk proteins are recognized by sRAGE (Liu et al. 2016; Zenker et al. 2019) as well as with the previously published data showing the affinity of CML‐modified β‐lactoglobulin to RAGE (Zenker, Teodorowicz, et al. 2020). Noticeably, no sRAGE inhibition is observed for WPH 2 which does not contain components of > 100 kDa. This is explained by the higher degree of hydrolysis, which leads to the absence of large peptides as well as an extra filtration step during the production process. This indicates a close relation between the processing conditions and the immunogenicity of the hydrolysate, and suggests that the reduction of glycation‐induced aggregates may lead to improved hydrolyzed formulas.

Recently we showed in another study that heat‐induced and glycation‐induced aggregates of β‐lactoglobulin (BLG) are recognized by sRAGE, Galectin‐3 receptor, and two receptors from the scavenger family: CD36 and SR‐AI (Teodorowicz et al. 2021). However, only glycated aggregates retained their binding to sRAGE and Galectin‐3 after digestion, demonstrating significantly higher binding compared to non‐glycated BLG (Teodorowicz et al. 2021). In line with the previous study on BLG as a model protein, the current study shows that high MW fractions isolated from hydrolyzed whey proteins bind to sRAGE. RAGE has been shown to play an important role in a number of inflammatory pathological conditions including systemic but also local intestinal and airway inflammation (Palanissami and Paul 2018; Oczypok et al. 2015). In murine models of asthma and allergic airway inflammation, RAGE has been shown to promote the expression of the type 2 cytokines IL‐5 and IL‐13 (Milutinovic et al. 2012), however, the direct link between RAGE and the development of food allergy has not been well documented yet. Smith and colleagues proposed in their false alarm hypothesis that AGEs present in the diet contribute to the development of food allergies via interaction with RAGE, which consequently primes immune signaling (Smith et al. 2017). Moreover, Ullah et al. (2014) and colleagues have shown that the HMGB1‐RAGE axis mediates allergic airway sensitization and airway inflammation. Given the fact that RAGE is highly expressed on antigen‐presenting cells, this receptor might be involved in allergic sensitization. We demonstrated enhanced IL‐1ß and IL‐8 levels after incubation of THP‐1 cells with fractions > 100 kDa isolated from WPH 1. It indicates the pro‐inflammatory character of this fraction which may be mediated via RAGE since the inhibition effect was observed after blocking of RAGE. The increased production of those inflammatory cytokines was not seen for the fractions with lower MW nor for WPH 2.

Lastly, the fraction of WPH 1 with MW higher than 100 kDa was shown to induce comparable basophil degranulation to non‐hydrolyzed WPC that was used as positive control. Fractions below 100 kDa induced significantly lower basophil degranulation compared to WPC. ß‐hexosaminidase release from RBLs with human FcεRI+ receptors has been previously reported to decrease upon prolonged hydrolysis of whey proteins. A large decrease in the release of ß‐hexosaminidase was observed after 15 min of hydrolysis (Knipping et al. 2012). Unseparated WPH 1 and WPH 2 led to ß‐hexosaminidase release of 55% and 10%, respectively, whereas the peptide fraction of < 3 kDa did not. This finding is in line with the results of Nutten et al. (2020) who characterize the MW profiles of commercially available EFHs showing that the content of potentially immunogenic peptides with MW > 1.2 kDa fluctuates between < 5% and > 15%. The presence of these larger peptides was positively correlated with BLG detection in ELISA as well as the capacity to lead to degranulation of IgE‐loaded RBL cells. Likewise, Vissers et al. (2011) previously demonstrated that the presence in the aggregates of multiple peptide epitopes/immunogenic structures in peanut protein extracts may facilitate IgE‐dependent receptor crosslinking and basophil release.

Based on the data presented here it can be concluded that partial—but not extensive—cow's milk protein hydrolysates can contain aggregated proteins/peptides. Those aggregates have the ability to induce basophil degranulation and activate innate immune cells expressing RAGE and other receptors that can bind to glycated or aggregated proteins. Next to that, our study showed a high complexity of partially hydrolyzed milk formulas, as comparing them within the same class of hydrolysates is challenging due to differences in aggregated protein content, peptide size, and peptide profiles. This indicates that partially hydrolyzed formula's should not be judged on functionality as a group, but rather based on the individual clinical efficacy of the single formula's.

## Author Contributions

All authors contributed to the design and implementation of the research. C.J.S. and M.T. performed the measurements, processed the experimental data, performed the analysis and designed the figures, under the supervision of R.J.J.v. N., M.L., and H.F.J.S. All authors participated in the discussion of the results and writing of the manuscript.

## Conflicts of Interest

R.J.J.v.N. and M.L. were employed by FrieslandCampina, Amersfoort, the Netherlands when the study was performed.

## Supporting information


Data S1.

## Data Availability

The data that support the findings of this study are available from the corresponding author upon reasonable request.
