# Supplementary material for: Functional Characterization of Glycated Peptide Aggregates in Whey Protein Hydrolysates
Source: Food Sci Nutr. 2025 Feb 19;13(2):e4704. doi: 10.1002/fsn3.4704 (PMC11836893; doi:10.1002/fsn3.4704)
Supplement: Supplementary file 1 — Data S1. [file FSN3-13-e4704-s001.docx]

**Supplementary materials**

Supplementary Figure 1. Efficiency of the fractionation analysed by SEC. Whey protein hydrolysates were fractionated by Filter Units, followed by fraction analysis by SEC. x-axis: fractions, obtained by fractionation on Filter Units; y-axis: results from SEC. Data have been normalized to 100%.

Human serum from milk allergic patients was received from Rijnstate Hospital (Arnhem, The Netherlands). Equal volumes of three different patients were pooled. Specific IgE levels were determined with ImmunoCAP (Thermo Fisher Scientific) and are presented in Table 1.

Supplementary Table 1. Specific IgE levels towards total milk and different milk proteins.

| **Patient** | **Cow’s milk [ku/L]** | **Casein [kU/L]** | **α-lactalbumin [kU/L]** | **ß-lactoglobulin [kU/L]** |
| --- | --- | --- | --- | --- |
| 1 | 65.5 | - | - | - |
| 2 | 328 | >100 | 33.4 | 30.4 |
| 3 | 2.62 | 1.58 | 1.58 | 0.72 |
